# Clinical Outcomes of Posterior Lumbar Interbody Fusion versus Minimally Invasive Transforaminal Lumbar Interbody Fusion in Three-Level Degenerative Lumbar Spinal Stenosis

**DOI:** 10.1155/2016/9540298

**Published:** 2016-09-26

**Authors:** Guoxin Fan, Xinbo Wu, Shunzhi Yu, Qi Sun, Xiaofei Guan, Hailong Zhang, Xin Gu, Shisheng He

**Affiliations:** Orthopedic Department, Shanghai Tenth People's Hospital, Tongji University School of Medicine, Shanghai, China

## Abstract

The aim of this study was to directly compare the clinical outcomes of posterior lumbar interbody fusion (PLIF) and minimally invasive transforaminal lumbar interbody fusion (MIS-TLIF) in three-level lumbar spinal stenosis. This retrospective study involved a total of 60 patients with three-level degenerative lumbar spinal stenosis who underwent MIS-TLIF or PLIF from January 2010 to February 2012. Back and leg visual analog scale (VAS), Oswestry Disability Index (ODI), and Short Form-36 (SF-36) scale were used to assess the pain, disability, and health status before surgery and postoperatively. In addition, the operating time, estimated blood loss, and hospital stay were also recorded. There were no significant differences in back VAS, leg VAS, ODI, SF-36, fusion condition, and complications at 12-month follow-up between the two groups (*P* > 0.05). However, significantly less blood loss and shorter hospital stay were observed in MIS-TLIF group (*P* < 0.05). Moreover, patients undergoing MIS-TLIF had significantly lower back VAS than those in PLIF group at 6-month follow-up (*P* < 0.05). Compared with PLIF, MIS-TLIF might be a prior option because of noninferior efficacy as well as merits of less blood loss and quicker recovery in treating three-level lumbar spinal stenosis.

## 1. Introduction

Degenerative lumbar spinal stenosis (DLSS) is the most common type of lumbar stenosis and increasingly being diagnosed in elderly with an approximate incidence of 5% in public [1]. Neurovascular structures compressed by the lumbar canal include various conditions, such as disc herniation, facet hypertrophy, bulging of the annulus, and thickening of the ligamentum flavum [2]. DLSS remains the most common indication for lumbar surgery in patients over 65 years old, and the main goal of surgical management is to decompress the spinal canal [3]. In addition, when the lumbar spine of DLSS patient is unstable, an instrumented fusion is usually recommended.

There were several surgical techniques available for decompression and augmented fusion, and each operative technique has its own merits and limitations. Posterior lumbar interbody fusion (PLIF) and transforaminal lumbar interbody fusion (TLIF) are two commonly used surgical techniques recommended for DLSS patients who fail conservative care to achieve spinal fusion [4]. Unlike the direct anterior approach, PLIF and TLIF reduce the risks of complications related to the vascular, abdominal, and reproductive systems [4]. Current indications for PLIF and TLIF include spondylolisthesis, degenerative scoliosis, severe instability, pseudarthrosis, recurrent disk herniation, and painful degenerative disk disease [4]. Long-term clinical results have confirmed the efficacy of PLIF and TLIF with high rates of fusion [5], and they all have the merit of adding anterior column support through a posterior only approach [4]. In addition, PLIF and TLIF were observed with better clinical outcomes than posterolateral spinal fusion alone in select patient populations [6].

Despite the established success of open PLIF and TLIF, open interbody fusions were observed with deleterious effects of prolonged paraspinal muscle retraction and extensive subperiosteal dissection [7]. These complications make it very difficult for surgeons to select an appropriate surgical procedure for multilevel DLSS. On the other hand, minimally invasive TLIF (MIS-TLIF) has become a popular technique for degenerative lumbar disease with merits of small incision, little lumbosacral muscle dissection scope, less bleeding, rapid recovery, and so forth [8]. In addition, many studies have confirmed that MIS-TLIF was associated with both cost saving and noninferior outcomes compared with an open approach [9–11]. Both MIS-TLIF and PLIF have been reported to be associated with favorable outcomes in treating DLSS, but we are not aware of any published literature directly comparing the clinical outcomes between open PLIF and MIS-TLIF for three-level DLSS. Therefore, the purpose of this study was to compare the clinical outcome of MIS-TLIF with PLIF in three-level DLSS.

## 2. Materials and Methods

### 2.1. General Information

The study was approved by the Local Institutional Review Board of Shanghai Tenth People's Hospital prior to data collection. Patients with spinal stenosis receiving three-segmental PLIF or MIS-TLIF in our hospital between January 2010 and February 2012 were included in this retrospective study. The investigators who conducted the data analysis were independent of the surgeons who conducted the surgeries. Basic characteristics of the participants such as age, gender, body mass index, and lesion segments were extracted and analyzed.

### 2.2. Surgical Techniques

**Figure 1:**
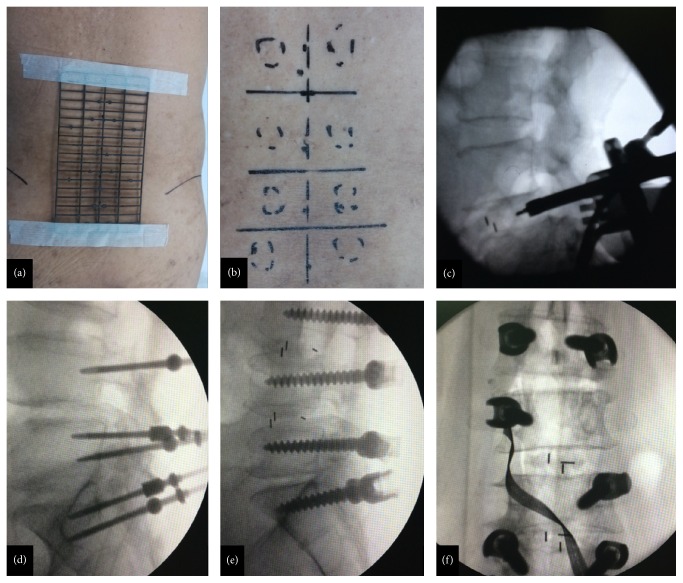
Surgical procedure of minimally invasive transforaminal lumbar interbody fusion. (a) Preoperative location with our self-made locator; (b) skin marker with the assistance of our self-made locator; (c) cage placement; (d) mark for screw placement; (e) lateral fluoroscopy of screw placement; (f) anteroposterior fluoroscopy of pedicle screw placement.

**Figure 2:**
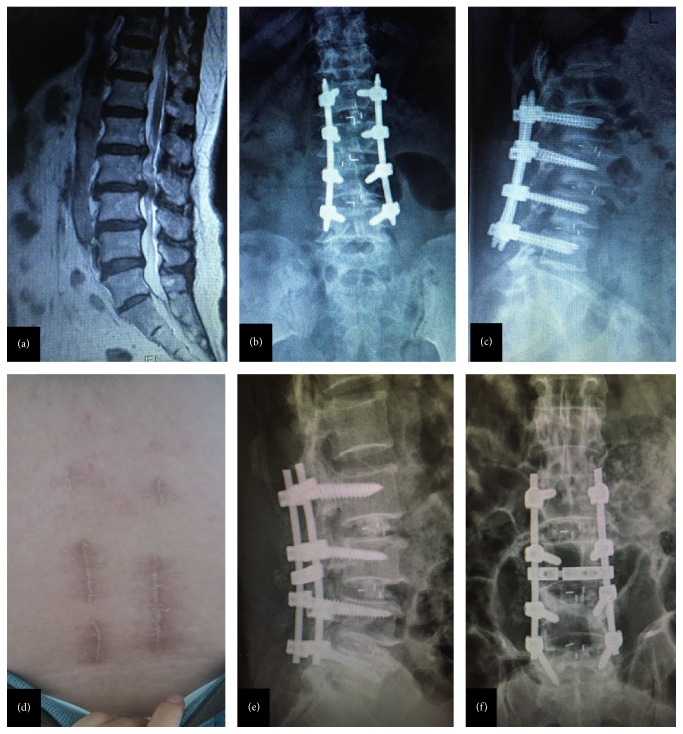
Minimally invasive transforaminal lumbar interbody fusion versus posterior lumbar interbody fusion in three-level spinal stenosis. (a) Preoperative magnetic resonance imaging; (b) anteroposterior fluoroscopy of three-level minimally invasive transforaminal lumbar interbody fusion; (c) lateral fluoroscopy of minimally invasive transforaminal lumbar interbody fusion; (d) minimal incision and skin healing of minimally invasive transforaminal lumbar interbody fusion; (e) lateral fluoroscopy of three-level posterior lumbar interbody fusion; (f) anteroposterior fluoroscopy of three-level posterior lumbar interbody fusion.

In MIS-TLIF group, all participants received general anesthesia before MIS-TLIF surgery. C-arm X-ray machine (Biplanar 500e; Swemac Medical Appliances AB, Sweden), METRx Quadrant System, and percutaneous pedicle screw (Sextant; Medtronic, Minneapolis, MN) were prepared before the operation. The patient was placed in a prone position on a radiolucent operating table. The waist bowed a bit, making the intervertebral space open and expanding Kambin triangle. The iliac crests were preoperatively palpated, and lines connecting to the uppermost margin of both iliac crests were marked (Figure 1). Under C-arm fluoroscopy, the targeted levels were confirmed according to our self-made locator. On the basis of the spatial relationship, the intervertebral spaces and the pedicle positions were marked on the body surface. An incision was planned by connecting a line between the outer portions of both ends pedicles (approximately 3.0 cm off midline). Then a skin incision about 3.0 to 4.0 cm was made on the more symptomatic side or more severe pathology side according to the imaging. The paravertebral muscles were split and retracted laterally to the outer edge of the facet joint, and the zygapophysis was confirmed. Expansion tube was then inserted and Quadrant System was placed. X-ray examination was repeated to confirm the target segments and the placement of Quadrant System. We conducted the decompression by cutting the inferior portion of the lamina, hypertrophied superior and inferior articular processes, and ligamenta flava. Then we enlarge the intervertebral space with distractor followed by PEEK cage (Capstone Medtronic Sofamor Danek, Memphis, TN, USA). After that, the percutaneous pedicle screw fixation was conducted (Figure 2).

In PLIF group, the patients were placed prone on the operating table after general anesthesia and tracheal intubation. After routine disinfection and draping, G-arm machine was used to confirm the targeted segment. Then a longitudinal incision was made in the middle of the spine, and muscular fasciae were cut apart. Musculus sacrospinalis were then bluntly dissected until lumbar transverse process was exposed. Pedicle screws were placed into the upper and subjacent vertebral pedicle of the segmental lesions. Spinous process, lamina, hyperplasia of yellow ligament, and interior zygapophysis were removed according to the scope of the lesions, and lateral recess as well as nerve root canal was enlarged with the protection of dural sac and nerve tissue. After that, fibrous rings were cut and nucleus pulposus was removed, and intervertebral space was open. The removed laminar and zygapophysis were crushed into smaller pieces for incorporation as autograft, and then the cage with crushed bones was also inserted. Next, titanium rods were used to connect the screws and fixed. G-arm machine was used to further confirm the pedicle screw placement and suture was conducted layer by layer after hemostasis. Finally, negative pressure drainage was placed and the incision was sewed up.

### 2.3. Clinical Assessment

The clinical outcomes were evaluated based on the improvement of back and leg pain, the disability, life quality, and the complications. Back and leg pain was measured using a ten-point visual analog scale (VAS) before surgery, postoperatively at six months and twelve months. Disability was assessed using the Oswestry Disability Questionnaire (ODI) before surgery and postoperatively at six months and twelve months. Health status of the patients was also evaluated with Short Form-36 (SF-36) scale before surgery and postoperatively at six months and twelve months. In addition, operating time, hospital stay, and the amount of blood loss during surgery were recorded, and the complications between the groups were also compared. All the above-mentioned measurements were routinely collected in our department as a research purpose, and we retrospectively analyzed these prospectively recorded data.

### 2.4. Statistical Analysis

The software package SPSS 12.0 (SPSS Corporation, USA) was used for statistical analysis. The statistic was demonstrated as mean ± SD. Independent student *t*-test was used to compare the difference of continuous variables between the two groups. Chi-square test was used to compare the difference of categorical variables between the two groups. *P* < 0.05 was regarded as statistical significance.

## 3. Results

In PLIF group, 17 patients were male and 19 were female with a mean age of 64.4 years. In the MIS-TLIF group, 14 patients were male and 10 were female with a mean age of 65.9 years. The lesion segments of L1–4 were 8 in PLIF group and 6 cases in MIS-TLIF group, while L2–5 were 16 versus 10 and L3-S1 were 12 versus 8 cases, respectively. In PLIF group, there were 13 cases with single-segmental fusion plus three-segmental fixation, 9 cases with two-segmental fusion plus three-segmental fixation, and 16 cases with three-segmental fusion plus three-segmental fixations. In MIS-TLIF group, there were 7 cases with single-segmental fusion plus three-segmental fixations, 8 cases with two-segmental fusion plus three-segmental fixation, and 9 cases with three-segmental fusion plus three-segmental fixations. The mean follow-up was 13.4 months for PLIF group and 14.2 months for MIS-TLIF group. There were no statistical significances in age, gender, lesion segments, fusion segments, and follow-up between the two groups (Table 1).

Radiographic examination did not detect any nonunion signs in both groups at final follow-up. Other clinical outcomes were demonstrated in Table 2. The operation time was 227.5 ± 17.1 min in PLIF group and 270.8 ± 33.7 min in MIS-TLIF group (*P* = 0.01). The estimated blood loss was 908.3 ± 242.9 mL in PLIF group and 666.7 ± 314.3 mL in MIS-TLIF group (*P* = 0.04). There were no significant differences in VAS of back pain between the two groups, either preoperatively or at 12 months after operation (*P* > 0.05). However, significant differences were observed when comparing preoperative VAS of back pain with 6 months or 12 months after operation (*P* < 0.05). Moreover, patients undergoing MIS-TLIF had significantly lower back VAS than those in PLIF group at 6-month follow-up (*P* = 0.03). Similarly, there were no significant differences in VAS of leg pain between the two groups, either preoperatively or at 6 months or 12 months after operation (*P* > 0.05). However, significant differences were observed when comparing preoperative VAS of leg pain with 6 months or 12 months after operation (*P* < 0.05). Additionally, there were no significant differences in SF-36 scores between the two groups, either preoperatively or at 6 months or 12 months after operation (*P* > 0.05). However, significant differences were observed when comparing preoperative SF-36 scores with 6 months or 12 months after operation (*P* < 0.05). Moreover, there were no significant differences in ODI between the two groups, either preoperatively or at 6 months or 12 months after operation (*P* > 0.05). However, significant differences were observed when comparing preoperative ODI with 6 months or 12 months after operation (*P* < 0.05). As for complications, 3 patients were found with cerebral fluid leakage in PLIF group, and one patient was found with cerebral fluid leakage and two with superficial surgical site infection in MIS-TLIF group (*P* > 0.05).

## 4. Discussions

The clinical outcome of MIS-TLIF compared with PLIF in three-level DLSS still requires clinical evidence. We retrospectively analyzed the patient-reported outcomes and identified that there were no significant differences between VAS of back pain, VAS of leg pain, SF-36 scores, and ODI at 12-month follow-up. To the best of our knowledge, this was the first clinical study with direct comparison of the clinical outcomes between PLIF and MIS-TLIF for three-level DLSS.

Lumbar interbody fusion is a well-validated technique with several different approaches such as anterior, lateral, transforaminal, and posterior approaches for a variety of conditions requiring spine stabilization [7]. Among them, PLIF is frequently used and may provide a higher immediate stability compared with that of MIS-TLIF, especially in lateral bending [12]. However, it may be limited by the thecal and nerve root retraction [13]. MIS-TLIF provides a minimal access through a paramedian approach with unilateral facetectomy, which offers the advantage of avoiding an anterior approach as needed for an anterior lumbar interbody fusion and limits the amount of neural retraction when compared to PLIF [14, 15]. There were a number of updated literatures supporting the use of MIS-TLIF with less intraoperative blood loss and decreased postoperative pain with lower overall complications rates [10, 11]. In addition, biomechanical analysis also demonstrated that MIS-TLIF, with one cage or two cages, provides reliable spinal stability [16]. However, in theory, PLIF may have the merits of adequate decompression over MIS-TLIF in three-level spinal stenosis. In contrast, patients undergoing MIS-TLIF may have quicker recovery and less blood loss due to minimal tissue injury. In practice, we did observe significantly less intraoperative blood loss and hospital stay of MIS-TLIF when comparing with PLIF. However, we did not observe lower overall complications rate, which might be due to the small sample size. Additionally, the VAS of back pain and leg pain were also significantly reduced and SF-36 were significantly improved after surgery at one-year follow-up. Furthermore, patients undergoing MIS-TLIF had significantly lower back VAS than those in PLIF group at 6-month follow-up, indicating MIS-TLIF might induce quicker improvement of back pain due to less tissue injury. Nevertheless, MIS-TLIF had significantly longer operation time due to more delicate surgical procedure.

Complications are nightmares for spine surgeons. The most common complications associated with PLIF and TLIF are intraoperative neurologic injury, interbody implant or bone graft migration, dural tear, surgical site infection, and so on. The overall reported complication rates of PLIF and TLIF range from 8% to 80%, which did not include potential pseudarthroses [17–21]. Mehta et al. found that neurologic injury was higher with PLIF than with TLIF (7.8% and 2%, resp.) [22]. Implant migration may be uncommon but very tricky. Aoki et al. [23] reported a series of three patients with posterior migration following TLIF. Dural tear or cerebral fluid leakage is a common complication whether during classic PLIF, open TLIF, or minimally invasive TLIF, which varies from 2% to 14% [24]. As for surgical site infection, it is reported in zero to 9% of patients [25]. We did not observe intraoperative neurologic injury, interbody implant, or bone graft migration, but three patients in PLIF group were found with cerebral fluid leakage and one patient was in MIS-TLIF group. Another two patients were identified with superficial surgical site infection in MIS-TLIF group but were all cured by routine administration of antibiotics. This condition might be due to the longer operation time in MIS-TLIF group and the bias from the small sample, although we admitted numerous factors such as exposure area and operation manners might also contribute to surgical site infection. However, we should also notice that the difference in infection rate between MIS-TLIF and PLIF group was not significant. Anyway, a prospective randomized control study with more subjects and longer follow-up would be in need to clarify this issue.

## 5. Conclusions

Current evidence indicated that MIS-TLIF was equivalent to PLIF in treating three-level lumbar spinal stenosis. Moreover, MIS-TLIF has the merits of less blood loss, quicker improvement of back pain, and shorter hospital stay. Prospective, randomized control study with larger participants and longer follow-up is needed to confirm this evidence.

## Figures and Tables

**Table 1:** Clinical characteristics of included patients with three-level spinal stenosis.

Variables	PLIF	MIS-TLIF	*P* value
Age	64.4	65.9	0.387
Gender			
Male	17	14	0.4
Female	19	10	
Lesion segments			
L1–4	8	6	0.96
L2–5	16	10	
L3-S1	12	8	
Fusion + fixation			
Single-segmental fusion + three-segmental fixation	13	7	0.75
Two-segmental fusion + three-segmental fixation	9	8	
Three-segmental fusion + three-segmental fixation	14	9	
Follow-up	13.4	14.2	0.58

**Table 2:** Clinical outcomes of PLIF versus MIS-TLIF in three-level spinal stenosis.

Parameters	PLIF (*n* = 36)	MIS-TLIF (*n* = 24)	*P* value
Operation time (min)	227.5 ± 17.1	270.8 ± 33.7	0.01
Blood loss (mL)	908.3 ± 242.9	666.7 ± 314.3	0.04
Hospital stay	15.5 ± 1.6	12.5 ± 2.8	0.00
VAS of back pain			
Preoperative	6.1 ± 1.1	5.9 ± 1.1	0.63
6 months after operation	2.9 ± 0.8^*∗*^	2.5 ± 0.9^*∗*^	0.03
12 months after operation	2.0 ± 0.7^*∗*^	1.7 ± 0.8^*∗*^	0.17
VAS of leg pain			
Preoperative	6.1 ± 1.0	5.8 ± 1.4	0.48
6 months after operation	2.7 ± 1.01^*∗*^	2.3 ± 1.0^*∗*^	0.18
12 months after operation	1.6 ± 0.7^*∗*^	1.2 ± 1.0^*∗*^	0.12
SF-36			
Preoperative	41.3 ± 3.6	43.4 ± 4.8	0.06
6 months after operation	57.8 ± 2.7^*∗*^	58.3 ± 2.0^*∗*^	0.44
12 months after operation	61.7 ± 2.6^*∗*^	62.39 ± 1.8^*∗*^	0.32
ODI			
Preoperative	37.8 ± 1.5	38.2 ± 1.9	0.35
6 months after operation	18.6 ± 1.7^*∗*^	19.3 ± 1.6^*∗*^	0.14
12 months after operation	18.4 ± 1.1^*∗*^	18.7 ± 1.1^*∗*^	0.33
Complications			
Surgical infection	0/36	2/24	0.08
Cerebral fluid leakage	3/36	1/24	0.53

^*∗*^ *P* < 0.05 versus preoperative.
